# Friendship networks and psychological well-being from late adolescence to young adulthood: a gender-specific structural equation modeling approach

**DOI:** 10.1186/s40359-016-0143-2

**Published:** 2016-07-11

**Authors:** Alexander Miething, Ylva B. Almquist, Viveca Östberg, Mikael Rostila, Christofer Edling, Jens Rydgren

**Affiliations:** Department of Sociology, Stockholm University, SE-106 91 Stockholm, Sweden; Centre for Health Equity Studies (CHESS), Stockholm University/Karolinska Institutet, SE-106 91 Stockholm, Sweden; Department of Sociology, Lund University, SE-221 00 Lund, Sweden

**Keywords:** Social network, Psychological well-being, Friendship network quality, Late adolescence, Young adulthood, Gender, Structural equation modeling, Two-wave panel data, Sweden

## Abstract

**Background:**

The importance of supportive social relationships for psychological well-being has been previously recognized, but the direction of associations between both dimensions and how they evolve when adolescents enter adulthood have scarcely been addressed. The present study aims to examine the gender-specific associations between self-reported friendship network quality and psychological well-being of young people during the transition from late adolescence to young adulthood by taking into account the direction of association.

**Methods:**

A random sample of Swedes born in 1990 were surveyed at age 19 and again at age 23 regarding their own health and their relationships with a maximum of five self-nominated friends. The response rate was 55.3 % at baseline and 43.7 % at follow-up, resulting in 772 cases eligible for analysis. Gender-specific structural equation modeling was conducted to explore the associations between network quality and well-being. The measurement part included a latent measure of well-being, whereas the structural part accounted for autocorrelation for network quality and for well-being over time and further examined the cross-lagged associations.

**Results:**

The results show that network quality increased while well-being decreased from age 19 to age 23. Females reported worse well-being at both time points, whereas no gender differences were found for network quality. Network quality at age 19 predicted network quality at age 23, and well-being at age 19 predicted well-being at age 23. The results further show positive correlations between network quality and well-being for males and females alike. The strength of the correlations diminished over time but remained significant at age 23. Simultaneously testing social causation and social selection in a series of competing models indicates that while there were no cross-lagged associations among males, there was a weak reverse association between well-being at age 19 and network quality at age 23 among females.

**Conclusions:**

The study contributes to the understanding of the direction of associations between friendship networks and psychological well-being from late adolescence to young adulthood by showing that while these dimensions are closely intertwined among males and females alike, females’ social relationships seem to be more vulnerable to changes in health status.

## Background

The importance of supportive social relationships has been confirmed for a wide range of health-related outcomes [1] and across various stages of the life course [2]. In late adolescence, associations with depression, psychological complaints and reduced psychological well-being have been documented [3, 4]. Depressive symptoms represent the most prevalent health problem in that age group in Sweden and other Western societies, particularly affecting females [5–8]. In Sweden, rates of psychological symptoms during late adolescence have increased continuously during the last three decades, especially among females [9, 10]. A poor sense of well-being has been identified as a marker of more severe subsequent psychological problems: Some studies posit that emotional problems and reduced well-being experienced in adolescence may remain as chronic health problems and thus persist into and beyond young adulthood [11–13]. It has also been shown that reduced well-being is associated with a subsequent higher risk of depression, self-harm, substance abuse, and suicide [14].

The transition from late adolescence to young adulthood commonly marks a significant shift in young people’s lives that includes leaving school and the parental home, as well as the engagement in new social contexts such as higher education or the labor market. During this period young people encounter new demands and responsibilities and are thus likely to experience increasingly more stressful situations. Friendships and social networks serve as important sources of social support that may help individuals to deal with the challenges that adult life entails and to alleviate the perception of stress [15, 16]. Intense social interaction and high-quality friendships may increase the ability to adjust to new social environments [17]. In this respect, friendships fulfill functions that family members often cannot adequately supply [17]. Moreover, successful social relationships enhance the individual’s capacity to socialize and build further social contacts [17–20] and are thus considered protective against maladjustment [21].

The quality of friendships changes over the life span and tends to rise with increasing age. Adult-like high-quality friendships – characterized by support, reciprocity, and intimacy – do not evolve until adolescence, and they become even more important as the individual enters young adulthood [22, 23]. It has been shown that various aspects of friendship quality are correlated with mental health outcomes in middle and late adolescence [24]. Scholars have suggested that low-quality relations and the lack of positive interaction may elicit anxiety, which in turn affects the adolescents’ social skills [19, 25]. The inhibited social functioning of the individual may then provoke withdrawal from peers that worsens well-being and leads to a further deterioration in social skills over time [19]. This cycle of bi-directional events – already emerging in childhood and becoming more manifest during adolescence [26] – makes it difficult to disentangle the causes and consequences in the association between the individual’s conditions and conditions in the peer group. A majority of past studies has maintained that induction is the predominant mechanism explaining the association between peer relations and mental health outcomes during adolescence [27]. The induction hypothesis suggests that peer groups influence the individual. Individuals tend to adapt to behaviors, norms, and attitudes extant in the peer group. This assumption, however, often relies on theoretical fundamentals rather than empirical evidence. In fact, at the near end of late adolescence, the direction of association appears to be reversed or at least reciprocal with both processes operating simultaneously: Due to older adolescents’ improved capacity to regulate peer influences, induction tends to decline while peer selection increases [27]. For example, an anxious person is thought to seek contact with anxious peers. This notion has been empirically confirmed by Borelli and Prinstein [28], who showed that depressive adolescents seek negative feedback from peer groups [28].

Gender is an important aspect to be considered in relationship processes during adolescence. As cognitive, emotional and behavioral development differs between adolescent males and females, their interactions with others and the way they form social networks vary. Adolescent females are usually more engaged in prosocial interactions [29] and are better able to develop supportive relationships with friends [12, 22, 30]. Females’ greater commitment and relational orientation may explain why they are better than males at mobilizing social support to master certain critical events [31]. However, earlier research demonstrated that males are more often found in disengaged peer groups, while females typically seek out higher commitment and relatedness in their best friendships [32]. As a consequence, females are thought to have a greater tendency to disrupt friendships [33] – partially because they tend to react more strongly to the violation of social norms in network relations. Therefore, friendship disruption and in particular the distortion of otherwise protective social ties may turn into a disadvantage and make females more vulnerable to depressive symptoms because they potentially reinforce the perception of stress and discomfort [33, 34]. In addition, co-rumination, the behavior of excessively discussing and revisiting problems with friends, is more common among girls than boys and may confound the benefits of close peer relationships [35, 36]. Females’ tendency towards stronger social commitments suggests that the association between network quality and psychological well-being is more positive among females than males. However, problematic social interactions and distortions in friendships may have adverse effects and wipe out the positive associations between network quality and well-being.

### Aim and research questions

The current study seeks to examine changes in friendship network quality and psychological well-being from late adolescence to young adulthood as well as the direction of associations of these relationships. Based on literature and theories discussed above, it is hypothesized that:

- H1: Friendship network quality and psychological well-being are positively correlated in late adolescence and young adulthood alike.
- H2: The association between friendship network quality and psychological well-being is more strongly pronounced among females, both in late adolescence and in young adulthood.
- H3: Friendship network quality in late adolescence influences psychological well-being in young adulthood, reflecting a process of social causation.
- H4: Psychological well-being in late adolescence influences friendship network quality in young adulthood, reflecting a process of social selection.
- H5: There are bi-directional associations between friendship network quality and psychological well-being from late adolescence to young adulthood, reflecting a reciprocal association between these dimensions.
- H6: The directionality of associations between friendship network quality and psychological well-being differs between females and males.

## Methods

### Data material

We use data from the Swedish survey *Social Capital and Labor Market Integration: A Cohort Study*, a two-wave survey on social capital and personal networks. The first wave of data collection was undertaken in 2009 and included a random sample of 2500 Swedish citizens born in 1990 to native parents. Thus, the vast majority of the respondents were 19 years of age at the time of the interview. The respondents completed a questionnaire through telephone interviews conducted by Statistics Sweden. The response rate was 55.3 % (*n* = 1382). Inaccessibility was a major cause of non-response – an issue related to the widespread use of unregistered prepaid phones in this age group – and to a lesser extent an unwillingness to participate. The non-response rate was somewhat higher among males and among those who lived outside the metropolitan areas. It was also higher among individuals who had not finished upper secondary school and had lower school marks, as well as among those whose parents had a lower educational level [37]. The second wave of data collection was carried out in 2013, i.e., when most respondents were 23 years old. Of the initial sample, 43.7 % responded to the questionnaire. The non-response pattern in terms of sociodemographic factors was similar to the first wave. The data material used in the current study is restricted to those individuals who participated in both waves and had full information on all study variables (*n* = 772). Compared to those who opted out, the remaining respondents had friendship networks of higher quality and better psychological well-being.

### Friendship network quality

The interview contained questions about friendship networks. The respondents were asked to think of the five persons (referred to as ‘alters’) with whom they spend most of their spare time. In a clarifying statement, respondents were asked to think of this as ‘friendship’. At Time 1 (T1), 8.3 % of the alters were family members or romantic partners, whereas this figure had increased to 16.6 % at Time 2 (T2). The distribution of the number of named alters was the following (here displayed as T1-T2): five alters, 58–56 %; four alters, 15–14 %; three alters, 18–19 %; two alters, 8–6 %; and one alter, 2–2 %. Acknowledging that peers may play multiple roles, and also that relatives and romantic partners may act as friends, all named peers were retained for the analysis. The respondents were subsequently asked about each one of their named alters, including a question related to the quality of the relationship: “*How good do you think your relationship is?*” There were five response options, ranging from ‘Not good at all’ (one point) to ‘Very good’ (five points). The measure of friendship network quality was derived by dividing the total number of points by the number of named alters. Thus, the measure indicates the average value of relationship quality within the network.

### Psychological well-being

Six indicators of psychological well-being were included in the current study, namely: “*I’m often tense and nervous*” (‘Tense’); “*I often feel sad and down*” (‘Sad’); “*I manage to do a lot*” (‘Energy’); “*Overall, I’m happy*” (‘Happy’); “*I’m mostly satisfied with myself*” (‘Pleased’); and “*I’m often grouchy or irritated*” (‘Grouchy’). The response options were: ‘Matches exactly’ (1); ‘Matches roughly’ (2); ‘Neither matches nor does not match’ (3); ‘Matches poorly’ (4); and ‘Does not match at all’ (5). For the analysis, the response options for the positive statements were reversed. Hence, a higher value for any of the six items indicates better psychological well-being. When all six items were included in an exploratory factor analysis (EFA) with varimax orthogonal rotation (performed separately for each combination of gender and wave of data collection), only the one-factor solutions provided eigenvalues above one. The rotated factor loadings for the one-factor solutions ranged from .40 to .66 for males and from .45 to .73 for females at T1 whereas they ranged from .39 to .72 for males and .39 to .69 for females at T2 (Table 1). Moreover, Cronbach’s alpha was .73 for males and .77 for females at T1, and .77 for males and .72 for females at T2.

**Table 1:** Factor loadings from exploratory factor analysis

	Males		Females	
	Factor 1	Uniqueness	Factor 1	Uniqueness
Time 1				
Tense	0.47	0.78	0.56	0.68
Sad	0.66	0.57	0.73	0.47
Energy	0.52	0.73	0.50	0.75
Happy	0.60	0.64	0.67	0.55
Pleased	0.65	0.58	0.64	0.59
Grouchy	0.40	0.84	0.45	0.79
Time 2				
Tense	0.52	0.73	0.46	0.79
Sad	0.71	0.50	0.69	0.53
Energy	0.58	0.67	0.48	0.77
Happy	0.68	0.53	0.64	0.59
Pleased	0.72	0.48	0.59	0.65
Grouchy	0.39	0.85	0.39	0.84

## Results

The distribution of friendship network quality and psychological well-being can be seen in Table 2. Note that higher values consistently correspond to higher friendship network quality and psychological well-being throughout the table. Gender differences were tested by means of independent sample t-tests, which showed that females have significantly worse well-being compared to males in terms of being tense and nervous, feeling sad and down, and not being pleased with themselves, at T1 and T2 alike. Moreover, at T1 they more often report that they are grouchy and irritated. There were no statistically significant gender differences for the remaining items. With regard to changes from T1 to T2, the results from paired samples t-tests show that friendship network quality improved slightly across the two time points, although this change was only statistically significant for females. Among males there were statistically significant increases in being tense and nervous, feeling sad and down, as well as feeling less pleased with oneself. For females, the only statistically significant change was seen for feeling grouchy and irritated, for which the reporting decreased over time. Although not shown in Table 2, it should be noted that the corresponding gender differences and differences across time had been present also if the mean values of psychological well-being had been used (males T1: 4.20; females T1: 3.94; males T2: 4.11; females T2: 3.99). The gender differences were statistically significant at each time point. Moreover, the differences between T1 and T2 were statistically significant for males but not for females.

**Table 2:** Distribution of the study variables (*n* = 772)

	Males (*n* = 393)				Females (*n* = 379)				*Comparison males-females* ^a^	
	Min	Max	Mean	St. dev.	Min	Max	Mean	St. dev.	Mean diff.	T-test
Time 1										
Friendship network quality	2.0	5.0	4.34	.53	2.8	5.0	4.34	.48	.00	n.s.
Tense	1.0	5.0	4.09	.96	1.0	5.0	3.64	1.09	.44	***
Sad	1.0	5.0	4.40	.88	1.0	5.0	3.91	1.07	.49	***
Energy	1.0	5.0	3.99	.89	1.0	5.0	3.90	.83	.09	n.s.
Happy	1.0	5.0	4.41	.73	1.0	5.0	4.37	.78	.05	n.s.
Pleased	1.0	5.0	4.17	.83	1.0	5.0	3.85	.91	.32	***
Grouchy	1.0	5.0	4.11	.82	1.0	5.0	3.96	.87	.15	*
Time 2										
Friendship network quality	3.0	5.0	4.38	.49	3.2	5.0	4.41	.43	-.03	n.s.
Tense	1.0	5.0	3.93	1.08	1.0	5.0	3.65	1.15	.28	***
Sad	1.0	5.0	4.24	.98	1.0	5.0	3.88	1.07	.37	***
Energy	1.0	5.0	3.92	.99	1.0	5.0	3.92	.92	.00	n.s.
Happy	1.0	5.0	4.38	.76	1.0	5.0	4.41	.78	-.03	n.s.
Pleased	1.0	5.0	4.09	.84	1.0	5.0	3.89	.99	.19	**
Grouchy	1.0	5.0	4.11	.92	1.0	5.0	4.16	.87	-.05	n.s.
*Comparison T2-T1* ^b^										
	Mean diff.		T-test		Mean diff.		T-test			
Friendship network quality	.03		n.s.		.07		*			
Tense	-.16		**		.00		n.s.			
Sad	-.15		**		-.04		n.s.			
Energy	-.07		n.s.		.02		n.s.			
Happy	-.03		n.s.		.05		n.s.			
Pleased	-.08		†		-.04		n.s.			
Grouchy	-.00		n.s.		.20		***			

Note: higher values indicate better friendship network quality and psychological well-being (items ‘Tense’, ‘Sad’, and ‘Grouchy’ are reversed)

^a^ A positive difference value reflects that males are better off compared to females, whereas a negative difference value suggests the opposite

^b^ A positive difference value indicates an improvement over time, whereas a negative difference value reflects the opposite

*** *p* <.001, ** *p* <.01, * *p* <.05, ^†^ *p* <.10

The gender-specific associations between friendship network quality and psychological well-being across the two time points were analyzed by means of structural equation modeling (SEM), with maximum likelihood estimation. As a first step, a baseline model was constructed with auto-regressive paths (measuring stability over time) from friendship network quality at T1 to T2 and from the latent factor psychological well-being at T1 to T2. Moreover, correlations between friendship network quality and psychological well-being were added at T1 and T2, respectively. Based on modification indices for omitted paths in the baseline model, some error terms for the well-being items were allowed to correlate (details available upon request).

**Fig. 1:**
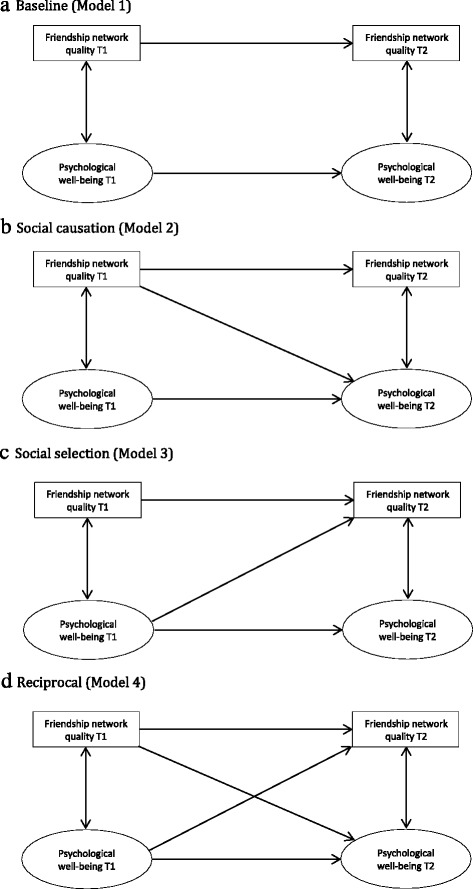
**a** Baseline (Model 1). **b** Social causation (Model 2). **c** Social selection (Model 3). **d** Reciprocal (Model 4)

Four competing models were subsequently tested, for males and females separately: the baseline model (Model 1); a social causation model, where friendship network quality at T1 predicts psychological well-being at T2 (Model 2); a social selection model, where psychological well-being at T1 predicts friendship network quality at T2 (Model 3); and a reciprocal model (Model 4). The models are illustrated in Fig. 1a–d.

A set of model fit statistics was derived for each of the four models: the Standardized Root Mean Square Residual (RMSEA), which should be below or close to 0.06 [38]; as well as the Comparative Fit Index (CFI) and the Tucker-Lewis Index (TLI), which both should be close to or above 0.95 [38]. The Akaike Information Criterion (AIC) and the Bayesian Information Criterion (BIC) were used to evaluate the relative goodness of fit, where lower values relative to the other models correspond to better fit [39]. Additionally, chi-square differences tests were performed to compare models that were hierarchically nested. The upper part of Table 3 shows the fit indices for males, whereas the lower part of the table gives the corresponding results for females. For males, all four models provide an acceptable fit to the data according to the values for RMSEA (.016–.018), CFI (.993–.995) and TLI (.991–.992). The baseline model (Model 1) has the lowest values for AIC and BIC. Moreover, the chi-square difference tests show that none of the other three models fit the data significantly better than Model 1. With regard to females, the values for RMSEA (.051–.052), CFI (.948–.950), and TLI (.929–.931) indicate acceptable fit of all four models. AIC is lowest for the social selection model (Model 3), whereas the baseline model (Model 1) has the lowest BIC. According to the chi-square difference tests, Model 3 fits the data significantly better than Model 1, on condition that *p* <0.10 is considered an acceptable level. It should be noted here that Model 4 provides a significantly better fit for the data (*p* = .08) compared to Model 2, but not compared to the other two models.

**Table 3:** Goodness-of fit statistics for the tested models (*n* = 772)

	Goodness-of-fit statistics			
	Model 1: Baseline^a^	Model 2: Social causation^b^	Model 3: Social selection^c^	Model 4: Reciprocal^d^
Males				
RMSEA	.016	.017	.018	.018
CFI	.995	.994	.994	.993
TLI	.992	.992	.991	.991
AIC	12178.444	12179.927	12180.417	12181.908
BIC	12381.108	12386.565	12387.055	12392.520
χ^2^	73.00	72.49	72.99	72.47
*df*	66	65	65	64
*p*	<.001	<.001	<.001	<.001
Chi-square difference test				
Comparison with:	-	Model 1	Model 1	Model 1/Model 2/Model 3
Change in χ^2^	-	.51	.01	.53/.02/.52
Change in *df*	-	1	1	2/1/1
*p*	-	.47	.87	.76/.89/.47
Females				
RMSEA	.051	.052	.051	.051
CFI	.949	.948	.950	.950
TLI	.929	.928	.931	.929
AIC	12115.777	12117.570	12114.797	12116.652
BIC	12316.592	12322.322	12319.549	12325.342
χ^2^	131.15	130.95	128.17	128.03
*df*	66	65	65	64
*p*	<.001	<.001	<.001	<.001
Chi-square difference test				
Comparison with:	-	Model 1	Model 1	Model 1/Model 2/Model 3
Change in χ^2^	-	.20	2.98	3.12/2.92/.14
Change in *df*	-	1	1	2/1/1
*p*	-	.65	.08	.20/.08/.70

^a^ Only auto-regressive associations and cross-sectional correlations

^b^ Friendship network quality at T1 predicts psychological well-being at T2

^c^ Psychological well-being at T1 predicts friendship network quality at T2

^d^ Friendship network quality and psychological well-being have reciprocal associations

**Fig. 2:**
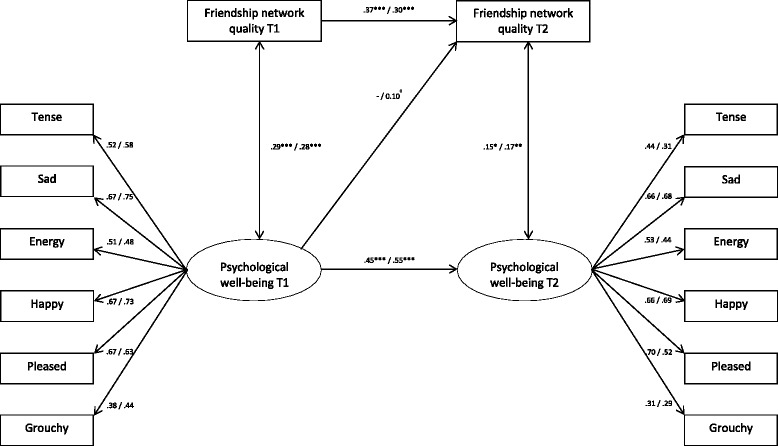
The associations between friendship network quality and psychological well-being (Males *n* = 393, Females *n* = 379). Results from structural equation modeling. Estimates (standardized) are displayed as males/females. *** *p* <.001, ** *p* <.01, * *p* <.05, ^†^ *p* <.10

Based on the model fit statistics, it was decided to proceed with Model 1 for males and Model 3 for females. The results from structural equation modeling are shown in Fig. 2 (for clarity, the error terms have been omitted from the figure). Concerning the measurement model, i.e., the latent factors representing psychological well-being, the included items show factor loadings of .38–.67 for males and .44–.75 for females at Time 1, and .31–.70 for males and .29–.69 for females at Time 2. It should be noted that a step-wise removal of the items with the weakest loadings did not significantly improve the model fit (data not presented).

With regard to the autoregressive paths, the coefficient for friendship network quality is .37 (*p* <.001) for males and .30 (*p* <.001) for females. For psychological well-being, the stability coefficients are .45 (*p* <.001) and .55 (*p* <.001) for males and females, respectively. Based on post-estimation procedure, it was shown that the stability of psychological well-being is significantly stronger than the stability of friendship network quality among females, whereas no such difference was found among males. Moreover, the correlation between friendship network quality and psychological well-being is .29 (*p* <.001) for males and .28 (*p* <.001) for females at Time 1, as compared to .15 (*p* <.05) for males and .17 (*p* <.01) for females at Time 2. For both males and females, post-estimation showed that the correlation is significantly weaker at T2 than T1. Finally, since the social selection model proved to have the best fit among females, the figure includes a path from psychological well-being at Time 1 to friendship network quality at Time 2. The coefficient of .10 (*p* <.10) suggests that better psychological well-being at T1 predicts an increase in females’ friendship network quality from T1 to T2.

## Discussion

The current study examines changes in friendship network quality and psychological well-being as well as the correlations between both measures across the transition period from late adolescence (age 19) to young adulthood (age 23). The use of structural equation modeling for longitudinal data made it possible to simultaneously investigate the social causation and social selection models of the association between network quality and well-being.

In line with prior research, the descriptive analysis demonstrated similar ratings of friendship network quality for males and females. Considering the transition into adulthood, females’ perception of their friendship network quality slightly improved. This finding confirms previous research which has demonstrated upward ratings of perceived friendship quality across late adolescence [24]. Also in line with past studies, the comparison of indicators of well-being showed significant gender differences, to the disadvantage of females, which persisted throughout the transition into young adulthood. Females’ salient high ratings on tenseness and sadness, for example, may further reveal their higher emotional vulnerability and reflect their increased propensity to suffer from psychological discomfort and depression [33, 40]. A comparison of the indicators of well-being between the two waves of data collection suggests that females’ overall well-being remains relatively stable during the transition from adolescence to adulthood whereas males exhibited higher levels of tenseness and sadness at age 23 compared to age 19. These elevated negative aspects among males may point to an increasing occurrence of stress and lack of coping capacities after adolescence [5]. Unlike young females, who accumulate coping skills during adolescence [33], young males may be less equipped with such abilities when entering adulthood. Despite the slight improvement in relationship quality among females as well as a decrease in well-being with regard to some specific items among males over time, both dimensions remained fairly stable.

Based on the results from structural equation modeling – also taking into account the autoregressive associations – friendship network quality and psychological well-being was found to be positively correlated at each separate time point, confirming H1. The similar correlations between network quality and well-being for males and females are remarkable, refuting H2 and deviating from the prevailing theories about females’ higher sensitivity to stress and difficulties in peer relationships [29]. It should also be noted that the correlation was significantly weaker at age 23 compared to age 19. From an induction perspective, this decrease in strength confirms previous research showing that peers exert a stronger influence on individuals during adolescence [41–43]. The weakened association may also underscore the stressful nature of adolescence [33] and hence adds support to the assumption that young people become increasingly resistant to peer influence when entering young adulthood [27]. Another explanation may be that young adults have developed more mature personality traits than 19-year-olds and have more settled life circumstances: 23-year-olds are about to complete their education and enter the labor market. Moreover, network settings of young adults are less rigidly structured compared to those of late adolescents. Whereas late adolescents’ social relations are still framed by parental influences and the school environment, adult individuals are more sovereign in interacting with others from various social settings and thus have more options to diversify their friendships networks [44].

Referring to H3, H4, and H5, the examination of cross-lagged associations aimed to show whether network quality in late adolescence predicted well-being in young adulthood and/or whether well-being in late adolescence determined network quality in young adulthood. Goodness-of-fit statistics showed that the baseline model without any cross-lagged associations provided the best fit for males. As a result, H3, H4, and H5 were rejected for males. The social selection model was most suitable for the female sample and suggested a weak cross-lagged association of well-being at age 19 on network quality at age 23, which confirms H4 for females. Given these results, we are also able to confirm H6, stating that there are gender differences in the directionality of associations between friendship network quality and psychological well-being. Drawing upon past research [33, 34], this may be because females whose well-being deteriorates over time are more likely to withdraw from or in other ways disrupt their social relationships.

Notwithstanding, the associations between well-being and network quality may be a result of third variable influences. For example, underlying health problems associated with well-being and relationship quality may have confounded the association or imposed a selection effect. Chronic health problems and pain, information about which was unavailable in the present study, have been shown to restrict peoples’ ability to participate in social activities [45]. As illustrated in Forgeron et al. [46], individuals with chronic pain tend to isolate rather than attempt to conceal their discomfort in social situations. Moreover, due to peers’ lack of empathy about what it means to suffer from pain and health constraints, adolescents with chronic health problems are more subject to rejection and victimization by peers than those without such impairments [46, 47]*.* Other relationship characteristics may also determine well-being or may have operated as a confounder in the presented analysis. However, an earlier study identified’relationship quality’ as strong determinant for well-being – being more important for well-being than other relationship aspects as for example trust to peers and self-disclosure [36].

### Strengths and limitations

A particular strength of this study is the unique data sample that surveyed ego-centric network data at two points in time, making it possible to account for cross-lagged and autoregressive pathways. The identified associations allow for conclusions about individuals in this particular age group, but it is reasonable to assume that they may also result in long-term consequences for the individuals’ mental health later on in adulthood.

Some drawbacks have to be noted despite the strengths of data and analytical methods. The non-response rates are relatively high because of the sampling procedure. The study sample was indeed positively selected, and if the respondents who were lost to follow-up had more health problems and less supportive networks than those who responded, that may have resulted in an underestimation of the associations at age 23. The model-fit comparison between the baseline model and social selection model showed a *p*-value of .08 in favor of the social selection model. This indicates a trend rather than significance and somewhat weakens our conclusion to proceed with Model 3. Moreover, some unobserved individual and network characteristics are potentially underestimated, which may have confounded the findings. However, the model specification already considers the relevant aspects that contribute to the associations of interest, i.e., the cross-lagged associations between relationship quality and well-being. The inclusion of additional control variables would impede meaningful interpretation of the model.

As the present study was based on self-assessed variables, the respondents’ misinterpretation of survey questions may have biased the outcomes. The same uncertainty also applies to structural aspects of peer relationships, such as the number of nominated alters. Although the number of friendships was taken into account when the measures were constructed, the respondents may have had more friends than they actually nominated. Limiting the possible number of peer nominations, however, restricts the study’s focus on significant social relationships and puts greater emphasis on close peers in individuals’ immediate social context. Friendship network quality was measured on a single dimension, with higher values representing higher relationship quality. Thus, the adverse effects from problematic relationships on well-being that were discussed in the introduction are not fully accounted for with this measure. Furthermore, accounting for average friendship quality may somewhat conceal the importance of high-quality friendships for well-being [48]. However, the present approach does acknowledge that female adolescents in particular have multiple friendships that provide a wider range of social support [49].

## Conclusions

By drawing on previous research on social causation and social selection, the study provided empirical support for the notion that associations between relationship quality and psychological well-being – at least in females – should be understood as result of cross-lagged associations between both dimensions.

This study has demonstrated that psychological well-being relates to perceived friendship quality, particularly in late adolescence. Although the correlation weakens when individuals enter young adulthood, the findings suggest that these dimensions should not be thought of as independent of each other. The mitigated association between network quality and well-being in young adulthood suggests that the importance of friendships for well-being decreases during the transition from late adolescence to young adulthood. Interventions that tackle reduced well-being and the increase of psychological symptoms would therefore seem more effective in adolescence than in adulthood. Moreover, despite the higher prevalence of low well-being among females, our findings suggest that the lack of high-quality friendships is associated with poor well-being among males as well. Still, we do not have sufficient knowledge about the causal pathways between networks and health, and whether these factors may differ by gender. Further research and policies focusing on these matters should therefore maintain a gender-specific approach when continuing to explore the interdependencies between psychological well-being and social network characteristics.
